# Weight Misperception, Self-Reported Physical Fitness, Dieting and Some Psychological Variables as Risk Factors for Eating Disorders

**DOI:** 10.3390/nu5114486

**Published:** 2013-11-13

**Authors:** Ignacio Jáuregui-Lobera, Mercedes Ezquerra-Cabrera, Rocío Carbonero-Carreño, Inmaculada Ruiz-Prieto

**Affiliations:** 1 Section of Nutrition and Bromatology, Pablo de Olavide University, Carretera de Utrera s/n, 41013 Seville, Spain; 2 Behavioral Sciences Institute, Fernando IV 24-26, 41011 Seville, Spain; E-Mails: informacionicc@gmail.com (M.E.-C.); inma.irp@gmail.com (I.R.-P.); 3 IES Atenea, Itaca 2, Mairena del Aljarafe, 41927-Seville, Spain; E-Mail: rociocarbonero@gmail.com

**Keywords:** weight misperception, self-reported physical fitness, diet, eating disorders, eating disordered behaviors, overweight, obesity

## Abstract

The aims of the current study were to explore possible gender differences in weight misperception, self-reported physical fitness, and dieting, and to analyze the relationship between these variables and others, such as self-esteem, body appreciation, general mental health, and eating- and body image-related variables among adolescents. In addition, the specific risk for eating disorders was examined, as well as the possible clusters with respect to the risk status. The sample comprised 655 students, 313 females and 342 males, aged 16.22 ± 4.58. Different scales of perceived overweight, self-reported physical fitness and dieting together with the Body Mass Index (BMI) were considered along with instruments such as the International Physical Activity Questionnaire (IPAQ), General Health Questionnaire (GHQ-28), Self-Esteem Scale (SES), Body Appreciation Scale (BAS) and Eating Disorders Inventory-2 (EDI-2). Since some gender differences were found with respect to these adolescent groups, it is necessary to design prevention programs that not only focus on traditional factors such as BMI or body image, but also on elements like weight perception, self-reported fitness and nutritional education.

## 1. Introduction

In the last few years, overweight-obesity during childhood and adolescence has risen and its stigma has worsened [1]. In Western countries—particularly how it has been studied in our Spanish context—there exists a negative perception of obese people [2]. Moreover, while obese people use positive adjectives to describe themselves more frequently, other groups tend to use adjectives with more negative connotations to describe those obese people [3]. Several studies have observed an association between overweight-obesity and negative psychosocial correlates and consequences [4,5,6,7,8,9,10]. A misperception of weight status has been defined as the discordance between an individual’s actual weight status and the perception of his/her weight status. Weight perception and misperception may influence the healthy or unhealthy behaviors people engage in [11]. In fact, misperception has repeatedly been documented among overweight and obese adults, and it has been hypothesized that weight misperception among overweight and obese individuals may preclude the adoption of healthful attitudes and behaviors, perhaps as a result of lower weight loss motivation. Overweight and obese individuals who consider their weight healthy, for example, might not try to lose weight and might be less inclined to eat healthfully and to be physically active [12]. On the other hand, some evidence indicates that weight misperception among overweight and obese individuals might be associated with healthful behaviors (e.g., better diet quality, more physical activity, and less sedentary behavior) [12,13]. Perceiving oneself as overweight-obese is relevant given the association between that perception and unhealthy weight-control behaviors [11]. Considering a high BMI, the misperception of overweight-obesity is a relevant but understudied field. Previous research suggests that overweight misperception varies according to gender—among other variables—as females tend to perceive themselves as overweight to a greater degree than males do, even at the same measured BMI [14,15,16]. Misperception of overweight-obesity among adolescents of normal weight might have negative consequences. The combination of overweight-obesity misperception, causing body dissatisfaction predicts dieting, and dieting is a clear risk factor for developing eating disorders [16]. In addition, adolescents who have been engaged in dieting and other unhealthy weight-control behaviors have been found to be at risk of weight gain over time [17,18].

With respect to different psychological and psychopathological variables, few studies have focused on the relationship between perceived overweight and self-esteem [19,20], as well as on the relationship between BMI and the measurement of a positive body image [21,22]. In general, even less research has been developed on perceived weight status which may also underlie the conflicting evidence on the relationship between classified overweight and its negative psychological correlates [23,24]. Not only depression and anxiety but also suicide ideation and attempts have been found to be associated with perceived overweight among adolescents [21,25,26,27,28].

Besides the concept of weight misperception, the awareness of one’s body shape or one’s body image plays a relevant role in different related behaviors [29]. Both weight misperception and poor body image have negative psychological effects such as low self-esteem, anxiety, depression and other psychosocial consequences (isolation, discrimination, family conflicts). It has been demonstrated that people engaged in a process of self-evaluation (included body checking) comparing themselves to others who they believe have more desirable sociocultural traits tend to be involved in behaviors aimed to achieve those desired characteristics [30].

Self-reported physical fitness is another variable to consider as a starting point to different healthy or unhealthy behaviors. Perceived physical ability (*i.e.*, the individual’s perception of physical abilities developed over time as a result of cumulative interactions with the environment) and perceived physical competence are two goal-oriented self-perception constructs [31]. Recently, it has been shown that body dissatisfaction is a significant mediator of the effect of BMI on perceived physical activity [32]. A large body of research has aimed to validate the idea that exercise improves body image through changes in physical fitness [33]. However, Martin and Lichtenberger [33] have suggested that improvements in physical fitness play a minor role in changing body image, because the effects of physical exercise and activity on body satisfaction should be mediated by changes in individuals’ perceptions of their physical fitness and competence. As a result, it seems that perceptions (weight, physical fitness) are core constructs to lead to healthy/unhealthy behaviors.

Based on the results of previous studies, the aims of the current study were: (1) to explore possible gender differences with respect to weight misperception, self-reported physical fitness and dieting; (2) to analyze the relationship between these variables and others like self-esteem, body appreciation, general mental health, and eating- and body image-related variables among adolescents; and (3) to examine the specific risk for eating disorders, as well as the possible clusters with respect to the risk status.

## 2. Experimental Section

### 2.1. Study Participants

The sample comprised 655 students, 313 females (47.80%) and 342 males (52.20%), with a mean age of 16.22 ± 4.58; they were all recruited from four schools in Seville (one public school and three charter schools, representing upper-middle socio-economical status) and were without any psychiatric history, which was assessed by means of a brief questionnaire at the time of obtaining the informed consent. None of the participants showed any comprehension and/or language difficulties or refused to participate. A total of 928 students were invited to take part in the study. Among them, 214 refused to participate and there were 59 students whose parents did not return the signed informed consent. Thus the response rate was 70.58%.

### 2.2. Instruments and Measures

#### 2.2.1. Perceived Overweight

As in a previous study, respondents were classified as “perceived overweight” if they responded “slightly overweight” or “very overweight” to the question “What do you think of yourself in terms of weight?” where other possible answers were “very underweight”, “slightly underweight” and “about the right weight” [34].

#### 2.2.2. Self-Reported Physical Fitness

Participants were asked about their physical fitness perception and they were classified as perceiving themselves as possessing a poor, fair, average, good or excellent physical fitness.

#### 2.2.3. Dieting

Participants were asked whether or not they were dieting at the moment (yes/no), the reason or reasons for going on that diet (aesthetic reasons, healthy reasons—other than losing weight—or only with the specific objective of losing weight), the origin of the diet (prescribed or self-imposed) and the intention to keep on dieting or being about to do it (yes/no).

#### 2.2.4. Body Mass Index (BMI)

As usual, BMI was calculated as the relationship between weight (in kg) and height squared (in m). Weight and height were taken in individual sessions, with the participants in the standing position, barefoot, and in light garments. A stadiometer “Añó-Sayol Atlántida S13” model was used.

#### 2.2.5. International Physical Activity Questionnaire (IPAQ)

There are several versions of this instrument according to the number of questions, the evaluation period and the method of application. The short version was used in this study, which provides information on the time spent on walking, moderate-intensity activities and vigorous and sedentary activities. Regarding the psychometric properties, the short IPAQ has shown about 0.65 reliability and adequate validity, having a reasonable agreement with the long form (*r* = 0.67, 95% CI: 0.64 to 0.70) [35].

#### 2.2.6. General Health Questionnaire (GHQ-28)

The Spanish version of this screening instrument of general psychopathology was applied. It has shown an adequate discriminative power (psychiatric case–no case) and it is easy to be administered. The questionnaire was designed to detect the presence of psychiatric cases in community and non-psychiatric clinical settings and comprises four seven-item scales: somatic symptoms, anxiety and insomnia, social dysfunction and depression. Each item consists of four possible answers, which are evaluated with 0 (the first two answers) and 1 point (the last two answers). Those evaluated with 0 points indicate absence of psychopathological problems, meanwhile those evaluated with 1 point indicate the existence of problems. By means of this scale of 0,0,1,1, the results are utilized to identify psychiatric cases. In this study, the recognition of possible cases was not the objective. This is why the final total score was used having in mind that a higher score indicates a greater psychopathology. The instrument has shown sensitivity between 76.90% and 84.60%, and specificity between 82% and 90.20% depending on the used cut-off points [36].

#### 2.2.7. Self-Esteem Scale (SES)

The scale comprises 10 items that are scored using a Likert format (from strongly agree to strongly disagree): the higher the score, the higher the degree of self-esteem. The Spanish version of the instrument shows adequate internal consistency (Cronbach’s a coefficient = 0.87), test-retest reliability (*r* = 0.72) and construct validity, and it was used in the current study [37,38].

#### 2.2.8. Body Appreciation Scale (BAS)

This scale is a 13-item instrument, whereby the factor structure comprises a single dimension and shows adequate internal consistency and construct validity, having proved to be useful for studying the positive aspects of body image. The Spanish version was used, which has shown an adequate internal consistency (Cronbach’s a coefficient = 0.91) [22,23].

#### 2.2.9. Eating Disorders Inventory-2 (EDI-2)

The EDI-2 is a self-report questionnaire comprising eleven subscales (drive for thinness, bulimia, body dissatisfaction, ineffectiveness, perfectionism, interpersonal distrust, interoceptive awareness, maturity fears, asceticism, impulse regulation and social insecurity). For this study, the Body Dissatisfaction (BD), Bulimia (B), and Drive for Thinness (DT) scales were administered. The BD subscale measures dissatisfaction with the overall shape and size of those parts of the body most related to eating disorders. The B subscale was designed to assess the tendency to think about and to engage in overeating episodes. The DT subscale measures excessive concern with dieting, preoccupation with weight, and fear of weight gain. The EDI-2 has been used to monitor psychological change during treatment of eating disorders, and the DT subscale has been used as a screening test. The internal consistency of the test, and its subscales, ranges between 0.83 and 0.92 in patient samples, and between 0.65 and 0.93 for various non-clinical samples. Test–retest reliability ranges between 0.41 and 0.97 depending on the sample [39].

### 2.3. Procedure

The study protocol was approved by the institution review board of the Behavioral Sciences Institute (Policlínica Los Remedios, Seville, Spain). After having obtained the schools’ headmasters’ permission and the students’ informed consent (as well as their parents’ approval), participants completed the aforementioned questionnaires and scales in group sessions without time limits. A psychologist, a nutritionist and a teacher supervised the procedure, instructing the participants about how to complete the questionnaires and scales until they were completely sure about their full understanding of the instructions. Data collection was developed in a suitable setting so the attainment of the task could be reached easily. All the participants volunteered to take part in the study and none of them received any kind of reward after fulfilling the task. The anthropometric measures were taken by four well-trained nutritionists with enough experience with working in this type of study.

### 2.4. Statistical Analyses

Data are expressed as means ± standard deviations. To study gender differences and others based on categorical variables, we considered the proportions (percentages), the analysis being done by means of χ^2^. An analysis of variance (ANOVA) was conducted to study differences with respect to the different variables included in the study, after having applied the Kolmogorov-Smirnoff test in order to analyze the data that fitted a normal distribution. Finally, a two-step cluster analysis was performed. It was agreed to apply the following three parameters: Log-likelihood distance measure (this default pre-cluster parameter is suitable for both continuous and categorical variables), a number of clusters automatically determined, and the Schwartz’s Bayesian clustering criterion. The software used for the analyses was IBM SPSS Statistics v20 for Mac (IBM-España, Madrid, Spain).

## 3. Results

### 3.1. Sample Description, Gender Differences and Risk of Eating Disorders

Participants were classified based on their percentiles (*P*) as: underweight (*P* < 5), normal weight (*P* between 5 and 84.9), overweight (*P* between 85 and 94.9), and obese (*P* > 95). When participants were 18 years old or higher, the classification based on the BMI was applied following the WHO guidelines [40]. There were 403 normal weight participants (61.50%), 152 underweight (23.20%) and 100 overweight or obese participants (15.30%).

There were no gender differences with respect to the weight perception scale. Overall, 41.22% of the participants misperceived their weight. Among those who perceived themselves as having a normal weight, but were slightly overweight or very overweight (*n* = 23), 18 were males (78.30%) (χ^2^ = 6.52; *p* < 0.01). On the contrary, among those who perceived themselves as slightly or very overweight but who did have a normal weight (*n* = 113), 67 were females (59.30%) (χ^2^ = 7.15; *p* < 0.01). Considering only the participants with a normal BMI (*P* between 5 and 84.9 and BMI 18.5–24.9), there was a significant gender difference with respect to the participants who perceived their status as overweight (slightly overweight and very overweight). In this case, 34% (67 out of 197) of females perceived themselves as overweight, while the percentage of males perceiving themselves as overweight was 22.40% (46 out of 205) (χ^2^ = 9.85; *p* < 0.01).

Taking into account the real weight and weight perception, among those participants who were underweight, 63.80% perceived their weight as being normal and 7.30% as being overweight or obese. Among the participants who were at normal weight, 28.10% perceived their weight slightly or very high and 5.90% considered their weight slightly or very low. Finally, among those who were overweight or obese, 59% considered themselves as at a normal weight and 24% perceived that they were slightly underweight (Table 1).

**Table 1:** Weight perception and actual BMI (%).

Weight Perception	Actual BMI		
	<18.5	18.5–24.9	>24.9
Very underweight	8.50	0.20	2.10
Slightly underweight	20.40	5.70	8.60
About the right weight	63.80	65.90	59
Slightly overweight	6.60	25.60	24
Very overweight	0.70	2.50	6.30

Based on the scores of IPAQ, 188 (28.70%), 386 (58.93%) and 81 (12.36%) participants were classified as having low, moderate and high level of physical activity respectively. With regards to the self-reported physical fitness, there were significant gender differences. While 36.90% of females considered their physical fitness as poor-fair and 24.30% considered it as good-excellent, in the case of males, these percentages were 20% and 43.40%, respectively (χ^2^ = 38.51; *p* < 0.001). Among those who had an actual low BMI, 45.77% considered that they had a good–excellent level of physical fitness. That percentage was 35.41% among those with a normal actual BMI and 14.13% for participants with a high actual BMI (χ^2^ = 68.33; *p* < 0.001). With respect to the scores on the IPAQ and considering low, moderate and high levels of physical activity, among those who had a low level, 28.47% perceived their physical fitness as good/excellent. When the level of physical activity was moderate, 43.05% perceived their physical fitness as good-excellent, the percentage being 46.66% in the case of those who had a high level of physical activity (χ^2^ = 28.66; *p* < 0.001).

With respect to going on a diet, among those who answered “yes”, 60.70% (51 out of 84) were females (χ^2^ = 8.63; *p* < 0.01); among those who were about to go on a diet based on aesthetic reasons, 62.40% (148 out of 237) were also females (χ^2^ = 38.72; *p* < 0.001).

There were 39 participants with a score on the EAT-40 ≥ 30 (5.95%). Among them, 5 (12.80%), 25 (64.10%) and 9 (23.10%) were underweight, normal weight and overweight, respectively. Considering their weight perception, 26 of them (66.60%) considered that they were slightly or very overweight. In the case of participants with a score on the EAT-40 < 30 that percentage was 28.10% (χ^2^ = 37.87; *p* < 0.001). In the group of participants with EAT-40 ≥ 30, 16 out of 39 participants (41.02%) perceived their weight as slightly or very high despite being normal, while in the group of participants with EAT-40 < 30 that percentage was 18.76% (χ^2^ = 16.31; *p* < 0.001).

With regards to the self-reported physical fitness, there were significant differences. While among participants with EAT-40 ≥ 30, 23 (58.97%) referred a poor–fair physical fitness, among the rest of participants, the poor-fair physical fitness were referred by 145 (28.10%) (χ^2^ = 31.18; *p* < 0.001). As far as the diet based on aesthetic reasons is concerned, 32 out of 39 participants with EAT-40 ≥ 30 (82.05%) claimed to be about to go on a diet. In the case of participants with EAT-40 < 30, this percentage was 41.90% (χ^2^ = 16.31; *p* < 0.001).

### 3.2. Weight Perception, Self-Reported Physical Fitness, Diet, Risk of Eating Disorders and Psychological Variables

Considering the weight misperception, the main differences were observed in the groups of low weight/weight perception as normal and normal weight/weight perception as high. In the first group, there were significant differences with respect to the GHQ-Chronicity (*p* < 0.05), as well as considering the age, BAS, EAT-40, SES, GHQ, BMI, self-reported BMI, EDI-DT, EDI-BD (*p* < 0.001). Thus, the first group obtained better results than the other groups using the different instruments (Table 2). In the second group there were significant differences with regards to the age, GHQ and BMI (*p* <0.05) as well as in BAS, EAT-40, SES, GHQ-Chronicity, self-reported BMI, EDI-DT and EDI-BD (*p* < 0.001). In this group the results obtained using the different instruments were worse than in the others (Table 3).

**Table 2:** Differences between participants who perceived their weight as normal (PNW) but who had an actual low weight (ALW) and the rest of individuals.

Instrument/Variable	PNW/ALW	Others
BAS	53.23	48.18 **
EAT-40	8.50	13.80 **
SES	31.56	33.12 **
GHQ	2.65	4.07 **
GHQ-Chronicity	12.85	14.11 *
BMI	17.04	22.16 **
BMI (self-reported)	17.86	21.94 **
EDI-DT	1.64	4.73 **
EDI-BD	2.06	5.00 **

* *p* < 0.05; ** *p* < 0.01.

**Table 3:** Differences between participants who perceived their weight as high (PHW) but who had an actual normal weight (ANW) and the rest of individuals.

Instrument/Variable	PHW/ANW	Others
BAS	42.32	50.29 **
EAT-40	18.09	11.23 **
SES	30.39	32.08 **
GHQ	4.73	3.68 *
GHQ-Chronicity	15.38	13.64 **
BMI	21.95	21.29 *
BMI (self-reported)	22.56	21.08 **
EDI-DT	7.16	3.70 **
EDI-BD	7.64	3.98 **

* *p* < 0.05; ** *p* < 0.01.

There were significant differences with respect to the several levels of self-reported physical fitness related to age, BAS, EAT-40, SES, GHQ, GHQ-Chronicity, BMI, self-reported BMI, EDI-DT and EDI-BD (*p* < 0.001). As far as the psychological variables are concerned, those groups that had poorer self-reported physical fitness produced worse results (Table 4).

The fact of going on a diet (or not) also led to some significant differences with respect to BAS, EAT-40, BMI, self-reported BMI, EDI-DT and EDI-BD (*p* < 0.001), as well as with respect to GHQ (*p* < 0.05). Those that went on a diet showed worse scores on variables such as body appreciation, eating attitudes, general mental health, drive for thinness and body dissatisfaction (Table 5). 

**Table 4:** Self-reported physical fitness and scores on the different variables included in the study.

Instrument/Variable	Self-Reported Physical Fitness				
	Poor	Fair	Average	Good	Excellent
Age	17.21	17.55	16.40	15.71	15.33
BAS	36.55	44.02	50.46	52.85	56.18
EAT-40	20.27	13.39	11.13	9.49	10.49
SES	28.30	30.23	32.34	33.66	34.97
GHQ	6.94	4.91	3.82	2.61	2.77
GHQ-Chronicity	17.42	15.54	14.15	11.82	11.21
BMI	24.60	22.82	21.26	20.34	19.39
BMI (self-reported)	24.20	22.76	21.08	20.26	19.99
EDI-DT	8.41	6.89	3.40	3.35	1.64
EDI-BD	11.86	7.56	3.69	3.03	1.55

In all cases *p* < 0.001.

**Table 5:** Differences between those who went on a diet and those who did not.

Instrument/Variable	Go on a Diet	
	Yes	No
BAS	45.79	49.96 **
EAT-40	18.36	10.54 **
GHQ	4.84	3.66 *
BMI	23.39	21.08 **
BMI (self-reported)	22.81	21.08 **
EDI-DT	7.53	3.75 **
EDI-BD	6.72	4.15 **

* *p* < 0.05; ** *p* < 0.01*.*

The group of EAT-40 ≥ 30 showed worse scores on BAS, SES, GHQ, GHQ-Chronicity, EDI-DT and EDI-BD (*p* < 0.001) than the rest of participants.

### 3.3. Cluster Analysis

In order to check possible different groups related to the risk of eating disorders, bearing in mind the variables included in this study, a two-step cluster analysis was performed. Log-likelihood distance measure, a number of clusters automatically determined, and the Schwartz’s Bayesian clustering criterion were applied.

As result, two different groups emerged, with 426 (65.04%) and 229 (34.96%) of the participants, respectively. There were significant differences between the two groups with respect to the scores on EAT-40, BMI, self-reported BMI and EDI-B (*p* < 0.05), as well as with regards to BAS, SES, GHQ, GHQ-Chronicity, EDI-DT and EDI-BD (*p* < 0.001). In all these variables the largest group emerges as a “high risk” group with higher scores on EAT-40, BMI, self-reported BMI, EDI-B, GHQ, GHQ-Chronicity, EDI-DT and EDI-BD, and with lower scores on BAS and SES. Considering the categorical variables included in the analysis, Table 6 shows the results.

**Table 6:** Categorical variables in the high/low risk groups after cluster analysis.

Variable	“High Risk” Group	“Low Risk” Group
Go on a diet for aesthetic reasons	Yes: 94.60%	No: 59.30%
Weight perception	Overweight: 60.70%	Normal: 81.50%
Self-reported physical fitness	Poor-fair: 50%	Average: 59.30%
Reasons to go on a diet	Lose weight: 62.50%	Health: 40.70%
Sex	Females: 64.30%	Males: 74.10%

## 4. Discussion

Summarizing our results, some gender differences emerge. Considering the participants with a normal BMI, 34% of females perceive themselves as overweight while in the case of males, that value is 22.40%. Overall, among those participants who are underweight, 63.80% perceive their weight at being normal. Thus the tendency to have a self-perception above the actual weight is frequent. The study of the self-reported physical fitness reveals another gender difference. While 36.90% of females consider their physical fitness as poor-fair and 24.40% consider it as good-excellent, in the case of males, these values are 20% and 43.40%, respectively. More differences occur with the frequency of dieting with 60.70% being female. The discrepancy between actual data and self-perceived status also appears with respect to the risk of eating disorders measured by means of the EAT-40. In this case, despite 64.10% of those who have a score on EAT-40 ≥ 30 have a normal weight, and 66.60% perceive themselves as slightly or very overweight. In addition, 58.97% consider themselves to have a poor-fair physical fitness while this value is 28.10% for the rest of the participants. As a result of the cluster analysis, we can identify a group of “high risk” individuals characterized by dieting for aesthetic reasons, with a high percentage perceiving themselves as overweight, with 50% self-reporting poor-fair physical fitness and 64.30% being female. Several studies have shown that females are more likely to misperceive themselves as overweight than males [12,13,14]. As a whole, our study does not find out gender differences in this regard. Nevertheless, some differences arose when considering the different self-perceptions. Thus, among those who perceive themselves as normal weight but who were actually overweight, males are predominant (78.30%). On the contrary the majority of participants who perceive themselves as overweight, but are of normal weight, are females (59.30%). These results make us think that men and women’s tendencies differ from one another: men tend to perceive themselves as normal weight in spite of being overweight, and women tend to perceive themselves as being overweight when they have a normal weight. In fact, among those who have a real normal weight, 34% of females perceive themselves as overweight; this percentage is only 22.40% in the case of males. Previous studies have shown values between 13.90% and 25.10% in the case of females, and between 7.40 and 8 in the case of males [34,41]. A comparison between real weight and weight perception should be considered as a risk factor for starting unhealthy behaviors. For example, the fact that being an obese or overweight person with the perception of having a normal weight could prevent those individuals from starting a healthier diet, taking up physical exercise, *etc.* This point has been highlighted previously [42]. The same applies in the case of individuals with normal weight perceiving that they are overweight leading to unnecessary dieting and weight loss. As a result of these types of findings, misperception of weight seems to be a usual finding with a double direction, which is to perceive a normal weight among overweight/obese people, and to perceive overweight/obesity among normal weight people. A comparison between the perceived weight and the measured BMI permits us to evaluate this weight misperception. Moreover, this weight misperception can be related to other psychological variables [41]. The self-perception of an individual as overweight has been linked to dieting and unhealthy weight-control behaviors (with the paradoxical effect of weight gain overtime), as well as disordered eating behaviors [17,18,37,43,44]. It must be noted that the self-reported physical activity is poorer in the case of females. In addition, among those who went on a diet, 60.70% were females, and among those who went on a diet for aesthetic reasons, 62.40% were also females. This constellation (a self-perception as being overweight while being normal weight, poorer self-reported physical activity and more dieting) would be a risk situation with regards to the development of eating disordered behaviors.

Taking into account the results of the group of participants who perceive their weight as normal, while having a low body weight, it seems that their psychological status is better when compared with other individuals. The perception of a normal weight seems to be more important than the actual weight considering different psychological variables as can be seen in Table 2. This is in comparison to those who perceive their weight as high, while it is actually normal. In this situation the psychological status results worse when compared with other participants (Table 3). Again, the weight perception seems to be more relevant than the actual weight with respect to the psychological well-being.

The importance of the perception not only lies with weight or body image but on the self-reported physical fitness too. The results shown in Table 4 suggest that the better that perception is, the better the global status of the participants will be. With respect to age it seems that the older the participants are, the worse self-reported physical fitness they have. A point to be considered is the fact that an actual low BMI is related with a good-excellent physical fitness, more than, for example, a normal BMI.

With respect to the specific risk of development of eating disorders as measured with the EAT-40, our results do not confirm other previous data in this regard. The current study shows that 5.95% of the participants have a score ≥ 30 on the EAT-40. Previous recent studies in the same context had shown percentages between 7.13% and 9.43% [45,46]. The most important fact seems to be that among participants with EAT-40 ≥ 30, 66.60% considered that they were overweight while this percentage was 28.10% in the case of participants with EAT-40 < 30. With respect to the weight misperception, more than 40% of participants with EAT-40 ≥ 30 and normal weight considered that they were overweight, this percentage being 18.76% in the case of individuals with EAT-40 < 30. In addition, participants with EAT-40 ≥ 30 showed poorer self-reported physical activity and more dieting especially for aesthetic reasons.

The psychological factors associated with weight perception, physical activity and diets are relevant. An association between overweight perception-misperception and psychological distress symptoms has recently been reported [36]. Our results confirm these data particularly in two groups: low weight/weight perception as normal and normal weight/weight perception as high. In these two groups lower scores on BAS and SES and higher scores on EAT-40, GHQ, GHQ-Chronicity, EDI-DT, EDI-BD as well as on the BMI and self-reported BMI were seen. These scores could be considered as a “worse psychological status”, which in turn is a risk situation among adolescents.

The cluster analysis emphasizes the notion of risk *vs.* protective factors with regards to eating disorders and eating disordered behaviors. The two emerging groups give us a picture about the high percentage of adolescents that could be at risk of disordered behaviors. Considering the relevance of the different predictors in order to classify the groups, the most relevant is the fact of dieting for aesthetic reasons. In the group of “high risk,” 94.60% responded affirmatively. BAS, EDI-BD, self-perception of weight, self-reported physical activity, other reasons to go on a diet, EDI-DT, SES, GHQ and GHQ-Chronicity were the most relevant variables to discriminate between the two groups. Also notable is that in the “high risk” group, 64.30% were females, while in the other group (“low risk”) 74.10% were males. The BMI and the self-reported BMI were higher in the case of the participants in the “high risk” group.

The limitation in interpreting the results of the cluster analysis lies in the fact that this is a cross-sectional study. Nevertheless, other studies have reported that weight perception may transcend actual body weight as a predictor of psychological distress and, in turn, of eating disordered behaviors [21,47,48,49,50]. In this regard, longitudinal studies should be developed bearing in mind that only a few factors seem to precede the onset of eating disorders. Another future line of study should be focused on the relationship between actual weight and self-perceived physical fitness. Having a low BMI associated with a good–excellent self-perceived physical fitness could be considered a risk for developing an eating disorder. That situation might prevent the individual from weight gain despite being underweight.

Engaging in unhealthy diets as a consequence of misperceived overweight has been described [24], so that overweight misperception and its relationship with some psychological distress (e.g., more general psychopathology, less self-esteem, less positive body image) could lead to clinical repercussions as a result of unhealthy diets. On the other hand, overweight individuals misperceiving themselves as having a normal weight could engage in unhealthy eating and perpetuate obesity-promoting behaviors [34]. More longitudinal studies need to be developed in the future in order to explore the connection between weight misperception and eating habits.

Considering the psychosocial variables in this field of study, self-esteem, depression, suicidal behavior, eating disorders and substance abuse, among others, have been studied [51,52,53,54,55]. Common risk factors that have been reported from longitudinal and cross-sectional studies on eating disorders are pregnancy complications, ethnicity, early childhood eating, feeding and gastrointestinal problems, sexual abuse and other adverse experiences, and general psychiatric morbidity. In addition, other risk factors included in the current study have also been reported previously such as gender, concerns about weight and shape, and negative self-esteem [56]. With respect to the specificity of those risk factors having been studied, the so-called putative risk factors [57], only a few of them have been reported to precede the onset of eating disorders, many factors being considered as general risk factors [58]. Nevertheless, few studies have been developed on the association between perceived overweight and self-esteem [34]. The same applies to the gap in the body image literature regarding the measurement of positive aspects, which has been pointed out by various authors [59,60].

As far as the age of the participants is concerned, the relationship between weight misperception and some of the psychological variables needs to be taken into account in the field of primary prevention of eating disorders. Misperception of overweight and other psychosocial variables contribute to the risk for disordered eating behaviors as we have found in a previous study [60].

## 5. Conclusions

Despite no gender differences being observed for the weight perception scale, as a whole, some specific differences seem to emerge if we take into account the combination actual weight/weight perception. In this case, misperception of being overweight seems to be more frequent in female adolescents, as has been reported previously in other studies. With regards to the self-reported physical fitness, there were significant gender differences, with a better perception among males. As far as dieting is concerned, females are more involved in that behavior, especially for aesthetic reasons.

The percentage of participants with EAT-40 ≥ 30 is significantly lower than the reported one in previous studies. Most of the participants with EAT-40 ≥ 30 perceive themselves as being slightly or very overweight.

Weight misperception, self-reported physical fitness, dieting and having an EAT-40 ≥ 30 are related to psychological variables such as body appreciation, self-esteem, general mental health and other specific eating disorder-related variables, such as a drive for thinness or body dissatisfaction.

The cluster analysis leads to the possibility of distinguishing two groups of adolescents with different risk statuses with respect to eating behavior. The larger group seems to be the one of higher risk. The idea of risk and protective factors needs to be studied in depth in future studies. In this regard, professionals and others who work with youth could promote prevention of specific eating disorders with programs highlighting factors such as those explored in this study: weight misperception, self-reported physical fitness and dieting along with other psychological factors such as self-esteem or general mental health.
